# Seasonality of Respiratory Viral Infections: Will COVID-19 Follow Suit?

**DOI:** 10.3389/fpubh.2020.567184

**Published:** 2020-09-15

**Authors:** Amani Audi, Malak AlIbrahim, Malak Kaddoura, Ghina Hijazi, Hadi M. Yassine, Hassan Zaraket

**Affiliations:** ^1^Department of Experimental Pathology, Immunology & Microbiology, Faculty of Medicine, American University of Beirut, Beirut, Lebanon; ^2^Faculty of Medicine, Center for Infectious Disease Research, American University of Beirut, Beirut, Lebanon; ^3^Biomedical Research Center and College of Health Sciences-QU Health, Qatar University, Doha, Qatar

**Keywords:** coronaviruses, COVID-19, severe acute respiratory syndrome coronavirus-2, respiratory viruses, seasonality, temperature, humidity

## Abstract

Respiratory viruses, including coronaviruses, are known to have a high incidence of infection during winter, especially in temperate regions. Dry and cold conditions during winter are the major drivers for increased respiratory tract infections as they increase virus stability and transmission and weaken the host immune system. The novel severe acute respiratory syndrome coronavirus-2 (SARS-CoV-2) emerged in China in December 2020 and swiftly spread across the globe causing substantial health and economic burdens. Several countries are battling with the second wave of the virus after a devastating first wave of spread, while some are still in the midst of their first wave. It remains unclear whether SARS-CoV-2 will eventually become seasonal or will continue to circulate year-round. In an attempt to address this question, we review the current knowledge regarding the seasonality of respiratory viruses including coronaviruses and the viral and host factors that govern their seasonal pattern. Moreover, we discuss the properties of SARS-CoV-2 and the potential impact of meteorological factors on its spread.

## Introduction

The severe acute respiratory syndrome coronavirus-2 (SARS-CoV-2) is the third zoonotic and highly pathogenic coronavirus (CoV) to emerge in the twenty-first century (1). The earliest cases of SARS-CoV-2 infections were reported in December 2019 in Wuhan, Hubei Province, China, the epicenter of the outbreak (1). Since then, the virus has been rapidly spreading across the globe (2).

CoVs are a large group of positive-stranded RNA viruses that commonly infect birds and mammals, causing a wide range of pathological conditions (3). These viruses undergo frequent mutations and recombinations, yielding new variants that can cross the species barrier (3). Since 1960, seven coronaviruses (CoVs) have been identified to cause infections among humans (4). Human coronaviruses (HCoV) 229E, OC43, HKU1, and NL63 are common in the human population and are responsible for about 15–30% of the annual respiratory tract infections (5). They are commonly associated with mild and self-limiting symptoms. Still, severe illnesses, accompanied by lower respiratory tract infection, might also occur, especially in elderly, neonates, and patients with underlying health conditions and risk factors (5).

In the current millennium, three highly pathogenic CoVs, SARS-CoV-1 (6), the Middle East respiratory syndrome CoV (MERS-CoV) (7), and the recently emerged SARS-CoV-2 (1), have crossed the species barrier and resulted in human infections. SARS-CoV-1 was first detected in Guangdong Province, China, in November 2002 and then rapidly spread to Hong Kong and 29 other countries, resulting in more than 8000 confirmed cases, including 774 deaths (6, 8). By July 2003, the virus died out throughout the world. MERS-CoV was first detected in Saudi Arabia in 2012, with the camels being the source for human infections (9). The virus caused a total of 2,519 laboratory-confirmed cases, including 866 associated deaths as of the end of January 2020 (7). The majority of cases were detected in the Kingdom of Saudi Arabia (KSA), in addition to one major outbreak in South Korea (10).

SARS-CoV-2 is a highly contagious virus that is associated with severe pneumonia cases (11). On January 30, 2020, the World Health Organization (WHO) announced COVID-19 (coronavirus infectious disease) as Public Health Emergency of International Concern after it affected 7,818 people with 170 deaths in 19 countries, including China (12). Since late February, the number of reported COVID-19 cases along with the number of affected countries had sharply increased within a short period, which led the WHO to declare the global COVID-19 outbreak a pandemic (13). Since then, the number of globally confirmed COVID-19 cases has been increasing exponentially, resulting in nearly more than 21 million confirmed cases and 761,000 fatalities as of August 16, 2020 (14).

Different approaches and interventions have been adopted to contain and control the disease spread including travel restrictions (global), partial or complete lockdowns (e.g., China and Singapore) (15, 16) and/or massive testing and isolation of confirmed cases and their contacts (South Korea) (17). The reluctance and delayed implementation of multilayered public health measures in some countries (e.g., Italy, Iran, the UK, Brazil, and the US) resulted in dire outcomes. Despite all the efforts and measures to contain the virus, it is still spreading globally, traversing all climate and environmental settings (2).

Nearly every acute viral disease has a particular seasonal window of occurrence, which differs according to the geographic location and environmental conditions (18). The incidence of respiratory viral infections is highly affected by seasonal changes, especially in temperate climates (19). Extensive research has been done to better understand the seasonality of respiratory viruses. Yet, our knowledge about this phenomenon remains limited. Here we attempt to address the possible impact of weather on SARS-CoV-2 spread, taking into consideration the current knowledge regarding its stability and transmission patterns, and the behavior of other respiratory viruses.

## Seasonality of Coronaviruses and Other Respiratory Viruses

Most viral respiratory infections tend to follow seasonal patterns with high incidence during winter in temperate regions and during the rainy season in tropical regions (20). Influenza virus and respiratory syncytial virus (RSV) have a single annual seasonal peak during winter in the Northern and Southern temperate regions (21). These viruses peak from December to March in the Northern hemisphere, and between June and August in the Southern hemisphere (21). Parainfluenza viruses have a seasonal peak from April to June in the Northern temperate sites and during September in the Southern temperate areas (21). In most of the tropical regions, these viruses occur year-round with increased incidence in rainy seasons (21, 22). Rhinoviruses and adenoviruses, two non-enveloped respiratory viruses, are known to circulate throughout the year in all climatic regions with occasional peaks in autumn and winter for rhinoviruses and in winter and early spring for adenoviruses (23, 24).

Epidemiologic studies of common cold HCoVs suggest that they exhibit a seasonal pattern. In a temperate climate, HCoV infections are primarily detected in winter and spring, with low-level circulation throughout the year (3). The early known types, HCoV-OC43 and HCoV-229E, predominantly circulate during the winter season in temperate climate countries (25, 26). An eight-year study of HCoV-OC43 and HCoV-229E among young adults in the US reported an equal number of infections with these two types during the winter (December through February) and spring season (March through May) (27). In Belgium, HCoV-OC43 and HCoV-229E were only detected in winter and early spring (28). Several other studies from the United States, Belgium, France, Canada, Japan, Jordan, Italy, and Germany consistently reported winter circulation of the other two HCoVs: NL63 and HKU1 (28–36).

On the other hand, tropical/subtropical regions display year-round circulation of HCoVs but with increased activity during certain months. A study conducted in China during 2008–2009 reported that HCoV-NL63 and HCoV-HKU1 infections, in hospitalized children with acute respiratory infections, showed increased activity during summer, fall, and winter (37). In another 7-year epidemiologic study between 2009 and 2016 in China, HCoVs circulated year-round but with the highest incidence during the spring and autumn (38). A study by Chiu et al., in Hong Kong, showed that HCoV-NL63 infections were notable during the spring and summer months of 2002, whereas HCoV-OC43 infections mainly occurred during the fall and winter of 2001 (39). Additionally, a study from Thailand confirmed the previous findings and reported the peak of HCoV-OC43 activity in winter, whereas HCoV-NL63 frequently occurred in autumn (40). In Australia, HCoV-NL63 peaks in mid-winter but was also detected between late-autumn and early-spring (41). Studies from some African countries (South Africa and Ghana) also reported a year-round circulation of HCoVs (42, 43).

Despite its rapid spread to about 30 countries, the SARS-CoV-1 was quickly contained. Thus, it was not possible to assess its seasonality. In the case of MERS-CoV, seven years have passed since its emergence and is still causing intermittent and sporadic infections without obvious seasonality (10). In fact, MERS-CoV has demonstrated low ability to transmit between humans, and most of the outbreaks have occurred mainly in healthcare settings. In camels, the virus seems to peak between late-winter and early-summer (44). This coincides with a spike in zoonotic transmission between April and July (45). A 5-year epidemiologic study, conducted between 2012 and 2017, demonstrated that MERS-CoV has the highest global seasonal occurrence during June with some observed seasonal variations (46). A case-cross-over analysis of the associations between primary human MERS cases and weather conditions found that the primary MERS infections are more likely to occur in cold and dry conditions (47).

In summary, most respiratory viruses follow a seasonal pattern. However, some factors might increase the incidence of these infections, even in seasons with low circulation. For instance, an increased incidence of respiratory infections occurs among pilgrims during the Hajj Season (48). The mass crowding in a limited space, in addition to the close contact between pilgrims, increases the risk of viral importation and transmission, particularly the respiratory ones (49) Rhinovirus, influenza virus, and the common cold HCoVs (mainly HCoV-229E) are usually the most commonly detected viruses during the Hajj (48).

## Drivers of Seasonality of Respiratory Viruses

Seasonality of viral respiratory infections can be primarily attributed to two main factors: the environmental and weather effects on the virus and the host, as well as the host's behavior and physiology (20). Studies on respiratory viruses, including influenza viruses, suggest that cold weather and low relative humidity are highly associated with the onset of respiratory infections in the temperate regions (50, 51). This was mainly attributed to the effect of temperature and humidity on the stability and transmissibility of the viral particles, in addition to the effect on the host airway immune response (19).

### Effect of Meteorological Factors on the Stability and Transmission of Respiratory Viruses

A study by Price et al. demonstrated that unlike the non-enveloped viruses that circulate throughout the year, enveloped viruses, including influenza and RSV, tend to be more seasonal, with a clear preference for colder temperatures (20). Harper et al. found that the optimal airborne influenza survival is at low temperatures and the survival decreases as the temperature increases (52). Low temperatures seem to enhance the lipid ordering of the viral envelope and improve influenza virus stability (53). This enhances the virus's ability to stay protected outside the body for a longer period of time (54). Further, a systematic review examined the factors that affect influenza survival on different metrics revealed that longer virus survival is favored at lower temperatures (55).

Besides their effect on stability, low temperature and relative humidity are also shown to enhance aerosol transmission of respiratory viruses (52). It was proposed that influenza virus transmission occurs mostly by aerosols in temperate regions and by contact in tropical sites (56). Using the guinea pig model, Lowen et al. showed that influenza virus aerosol transmission is suppressed by high humidity and warm temperature, but enhanced under cold and dry conditions (57). Low relative humidity induces evaporation of water from the exhaled bio-aerosols, leading to the formation of droplet nuclei (1–5 μm in size) (58). The extent of infectious viral particles survival in dried aerosols is not known; however, it is speculated that these nuclei can stay suspended in the air for prolonged periods (58). The opposite happens at high relative humidity, whereby the respiratory droplets increase in size by taking on water from the surrounding and quickly settle out of the air, thus, decrease aerosol transmission of the virus (58).

On the other hand, the transmission of influenza viruses by contact was shown to be efficient even at high humidity (54). High humidity enhances the indirect virus transmission by increasing the virus particle's stability, inside droplets, on surfaces (54). A study by Yang et al. showed that humidity promotes the survival of influenza A virus by controlling the extent of evaporation in these virus-containing droplets, which affect the solute concentrations and thus, viral stability (59). This partially explains the year-round occurrence of viral respiratory infections in tropical regions, particularly during rainy seasons when humidity is high.

In addition, it is well-known that solar UV radiation (UV) of all wavelengths effectively inactivate RNA and DNA viruses to varying extents (60, 61). Three types of UV radiations, UVA, UVB, and UVC, exist in nature, with UVC, having the shortest wavelength range, being the most effective against viruses (62). However, only UVA and UVB radiations are found at the ground-level sunlight, and these are known to have lower efficiency against viruses (60). The low incidence of respiratory infections during summer in temperate regions can also be attributed to the solar inactivation of viruses on the outdoor surfaces contaminated with respiratory secretions, thus decreasing the possibility of fomite transmission.

### Effect of Meteorological Factors on Host's Susceptibility to Infection

Meteorological or environmental conditions were also shown to have a direct effect on the host's susceptibility to infections (63). The role of cold weather in weakening the immune response is controversial (63, 64). However, many studies indicated that cold and dry environments have an immunosuppressive effect on the host, and thereby increase the risk of acquiring infections (65–67). Increased exposure to cold air was shown to induce a temperature-related reduction in lung function in patients with chronic inflammatory airway diseases, such as chronic obstructive pulmonary disease (COPD) and asthma (68). Seasonal changes in temperature were also shown to affect the local immune response in the nose (66). It was shown that the antiviral defense response against rhinovirus infection in cultured mouse airway cells is reduced at low temperature (69). The cooling of the nasal airway by the inhaled cold air induces a decrease in the temperature of the respiratory epithelium, and compromise both the mucociliary clearance (MCC) in the nose and the local immune response in the upper airway (66).

The nasal respiratory epithelium is made up of ciliated cells covered with an airway surface layer comprised of a mucus layer that catches inhaled particles and low viscosity pericilliary layer that moisturizes the surfaces and enable ciliary beating (70). MCC is a key mechanism required for getting rid of particles, including infectious agents, stuck on the surface of the respiratory epithelium (70). Production of thin mucosal layer and beating of cilia at a specific frequency are considered key factors for efficient MCC (66). MCC was shown to be affected by temperature and relative humidity. A recent study demonstrated that MCC and epithelial cellular repair in influenza virus-infected cells is reduced at a low relative humidity (67). Using a climate chamber for cell culture, a study showed that a temperature of 25°C and RH of 40% induced more production of mucin compared to 37°C and 80% RH (71). In addition, it was shown that tracheal and nasal mucociliary beat frequency decreases as the temperature falls below 20°C and totally ceases at 5°C (72). These studies indicate that low temperature and low humidity in the nasal airway compromise the MCC by increasing mucin secretion and reducing mucociliary beat frequency (67, 71, 73). Moreover, a study done on guinea pigs revealed that breathing dry air can disrupt cilia, damage epithelial cells, and induce local inflammation of the trachea (74). More importantly, the phagocytic activity of macrophages, a key non-specific immune response mechanism against viruses, was found to be reduced both *in vivo* and *in vitro* at low temperatures (75).

It has also been postulated that shortened exposure to sunlight during the winter affects vitamin D levels, a key modulator for both innate and adaptive immune responses, which increases the susceptibility to respiratory infections during winter (76, 77). A systematic review assessing the relation between vitamin D and respiratory tract infections found that vitamin D supplementation reduces the incidence of respiratory tract infections (78). The high incidence of influenza was also correlated with the seasonal decrease in vitamin D levels (79). A recent observational study of 212 patients from three South Asian hospitals, found a positive association between vitamin D levels and clinical outcomes of COVID-19 patients (80).

## Modes of SARS-CoV-2 Transmission

The respiratory transmission mode of SARS-CoV-2 is not fully understood. However, the virus is assumed to have a transmission pattern similar to that of the influenza virus (81). These modes include transmission through direct or indirect contact with infected individuals. Transmission of the virus can occur via fomites or direct contact with an infected person or through respiratory droplets released during sneezing, coughing, or talking (82). Studies showed that SARS-CoV-2 can stay viable on surfaces for hours or even days especially in healthcare facilities where the concentration of the virus released by the patients is relatively high (82–84). The survival of the virus on these surfaces depends on relative humidity and temperature and on the nature of the contaminated surfaces (85, 86).

Although airborne virus transmission has not yet been confirmed in humans, studies suggest that the occurrence of aerosol transmission cannot be excluded, especially in closed venues (82, 87). Airborne transmission occurs when the aerosols (droplet nuclei <5 μm) containing infectious viral particles spread in air over a long distance and remain suspended for a long time (82). These aerosols are produced from evaporation of large respiratory droplets or released from the infected individuals by coughing, sneezing, talking, or exhaling. The aerosols can be breathed by individuals and cause infection if enough infectious dose of the virus is present or upon extended exposure (82). A study by Van Dormalan et al. found that SARS-CoV-2 virus particles remained infectious for 3 h in experimentally generated virus-containing aerosols that mimic the human-generated ones (83). Several studies reported detecting SARS-CoV-2 RNA in the air samples collected from different areas inside the hospitals such as patients' toilet areas, medical staff areas, and public areas prone to crowding (83, 88, 89). Recently, it was shown that infectious SARS-CoV-2 can be detected in air samples collected 2–4.8 m away from hospitalized COVID-19 patients, supporting the possibility of airborne transmission at least in confined environments (90).

The possibility of transmission via the fecal-oral or fecal-respiratory route has been also considered for COVID-19. SARS-CoV-2 RNA and viable virus were also found in urine and feces of infected patients (91–95). However, no evidence on virus transmission through feces or urine exists (91–95). Some studies also reported the detection of SARS-CoV-2 RNA but not infectious virus in blood samples of COVID-19 patients and breast milk of infected mothers (91, 96, 97). The absence of viable virus in blood and breast milk excludes the possibility of virus transmission through these routes (91, 96, 97).

Controlling the transmission of respiratory viruses is very challenging on its own, but is even more complicated in the case of SARS-CoV-2 due to the well-demonstrated role of asymptomatic or pre-symptomatic carriers (98–101). A meta-analysis of nine studies from six countries (including 21,035 close contacts of 843 COVID-19 cases) estimated the proportion of asymptomatic COVID-19 carriers at 15% (95% CI 12–18%). The transmission rates ranged from 0 to 2.2% for aymptomatic cases compared to 0.8–15.4% among symptomatic ones (102). Lau et al. estimated the presymptomatic transmission proportion to be 44% (95% CI, 30–57%) with infectiousness peaking between 2 days before and 1 day after symptoms onset (103). While a study carried out in Singapore found that around 6.4 % of the secondary infections are caused by the pre-symptomatic patients (104).

## Role of Meteorological Factors During SARS-CoV-2 Transmission

The seasonal differences between the Southern and Northern hemispheres might have played a role in the spread of SARS-CoV-2. Early in the pandemic, Northern hemisphere countries with cold climates appeared to be the most vulnerable to COVID-19 transmission, while tropical regions and those in the Southern hemisphere seemed to be the least affected. Initial studies suggested a potential role for meterological factors in the spread of SARS-CoV-2. Sajadi et al. found more virus spread in areas with an average temperature of 5–11°C and absolute humidity of 4–7 g/m^3^, suggesting a potentially seasonal behavior (105). Another study found that around 90% of the cases were reported in countries with temperatures maxima below 17°C and absolute humidity of 3–9 g/m^3^. The study suggested that the summer season might reduce the impact of COVID-19 pandemic in those countries as the temperatures rise (106). Another study concluded that SARS-CoV-2 transmits more easily in countries with relatively cool conditions and that transmission is reduced in sites with high temperatures and high relative humidity (107). Chen et al. reported that the optimal temperature for virus spread was found to be at 8°C and humidity between 60 and 90%. The authors suggested that the weather plays a key role in the transmission of SARS-CoV-2 around the world (108).

The association between the daily incidence of COVID-19 cases and climatic conditions in mainland China was examined between January 20 and February 29, 2020. Using modified susceptible-exposed-infectious-recovered (M-SEIR) model, Shi et al. found that COVID-19 transmission rate decreased at higher temperatures (109). However, another study conducted during the same period in China concluded that the increase in humidity and temperature alone would not reduce the virus spread if the public health interventions have not been strictly implemented (110). Similarly, a prospective cohort study done on 144 different areas other than China, South Korea, Iran, and Italy, found that it is the strict interventions that are strongly associated with the decrease in virus transmission but not latitude and temperature (111). Nonetheless, the early lockdowns in some countries and variable public interventions taken by various countries hindered the ability of scientists to study the association between climate and virus transmission. The aforementioned studies are also being challenged by the fact that many countries in the Northern hemisphere are witnessing a second wave of COVID-19 despite entering the summer season.

## Conclusion: Will COVID-19 Become Seasonal?

The basic reproduction number (R0) is the number of secondary cases resulting from a primary case in a susceptible population and is an important indicator to predict the spread of a virus. For a virus to follow a seasonal pattern, and thus wane in summer, its effective R0 should drop below 1 (112). For SARS-CoV, the R0 is estimated between 2 and 3 (112) and in some estimates as high as 5.7 (113). As discussed above, several factors in the summer might reduce the effective R0 of respiratory viruses including the effect of warm tempertures and humidity on the stability of the virus and susceptibility of the host as well as behavior of the population such as indoor crowding. For seasonal influenza virus, its R0 is estimated to be 1.27 (114). Therefore, these aforementioned factors could easily drop the effective R0 to below 1 in summer halting the virus spread and resulting in the observed seasonal pattern of flu. The warm temperatures and humidity of the summer might impact the host immune response and thus its susceptibility to infection by SARS-CoV-2 similar to its effect on influenza (66). However, other factors including: (1) a much higher R0, (2) higher stability of SARS-CoV-2 (it can survive for up to 72 h on hard surfaces at temperatures between 21 and 23°C and in relative humidity of 40%) (83), and (3) a largely immunologically naïve population against SARS-CoV-2 compared to influenza make it unlikely for the R0 to drop in summer enough to halt the spread of SARS-CoV-2. Therefore, without public health interventions, SARS-CoV-2 will continue to spread in summer as witnessed in many countries around the world. Nonetheless, as the population herd immunity is attained through natural infections and/or vaccinations then the effective R0 is expected to drop substantially making the virus more prone to seasonal fluctuations.

## Author Contributions

HZ conceived the review idea and supervised the writing. AA developed the review outline and coordinated the drafting of the manuscript. AA, MA, MK, and GH wrote the manuscript. HY critically reviewed the manuscript. All authors revised and approved the final version of the manuscript.

## Conflict of Interest

The authors declare that the research was conducted in the absence of any commercial or financial relationships that could be construed as a potential conflict of interest.
